# Vaginal epithelioid trophoblastic tumor mimicking vaginal fistula: a case report and literature review

**DOI:** 10.3389/fonc.2025.1593126

**Published:** 2025-09-12

**Authors:** Dong-mei Li, Xiu-zhang Yu, Ming-rong Qie, Rui-qi Duan

**Affiliations:** ^1^ Department of Obstetrics and Gynecology, West China Second University Hospital, Sichuan University, Chengdu, China; ^2^ Key Laboratory of Birth Defects and Related Diseases of Women and Children (Sichuan University), Ministry of Education, Chengdu, China

**Keywords:** ETT, vaginal fistula, diagnosis, treatment, case report

## Abstract

**Background:**

Epithelioid trophoblastic tumor (ETT) is a rare variant of gestational trophoblastic neoplasia. This article presents a case of vaginal ETT, initially misdiagnosed as vaginal carcinoma, in a patient with no history of gestational trophoblastic disease. The aim is to explore the clinical characteristics and diagnostic features of this condition.

**Case presentation:**

A 50-year-old woman presented with a 3-year history of vaginal pain. Following a vaginal fistula repair at an external hospital, a biopsy unexpectedly revealed vaginal carcinoma, prompting referral to our institution for further management. Pathological examination confirmed a diagnosis of extremely rare vaginal ETT, with immunohistochemistry showing characteristic marker positivity. Notably, the patient had no history of gestational trophoblastic disease, and serum Human Chorionic Gonadotropin (HCG) levels remained normal throughout. After diagnosis, the patient underwent total hysterectomy, bilateral salpingo-oophorectomy, and partial vaginectomy. Postoperative pathology confirmed the primary site to be the vagina, an unusual location for ETT. To further control the disease, the patient received 6 cycles of EMA-CO chemotherapy. Follow-up at 1 year showed no recurrence or metastasis, with stable disease.

**Conclusion:**

ETT often present with nonspecific symptoms, which can lead to misdiagnosis. Vaginal delivery and induced abortion may be potential risk factors. Clinically, in patients presenting with vaginal pain, masses, or genital tract fistulas, the possibility of a trophoblastic tumor should be considered and thoroughly evaluated.

## Introduction

Epithelioid trophoblastic tumor (ETT) is a rare and distinct form of gestational trophoblastic neoplasia originating from intermediate trophoblasts of chorionic origin. First described in 1998, ETT arises from these intermediate trophoblasts (1). Retrospective studies indicate that vaginal bleeding is the most common clinical manifestation of ETT (2), with the lungs being the most frequent site of distant metastasis (3). ETT typically exhibits slow tumor growth, and most cases are confined to the uterus in the early stages, with metastasis being relatively uncommon. Although the precise pathogenesis of ETT remains unclear, existing research suggests a strong association with prior gestational events, particularly normal vaginal delivery, hydatidiform mole, and a history of abortion (4). Additionally, ETT shares histological features with cervical squamous cell carcinoma (5), and its nonspecific clinical presentation often leads to misdiagnosis (6). Due to the limited understanding of ETT, preoperative diagnosis is challenging, and definitive diagnosis is usually made only through postoperative pathological examination. This article presents the first reported case of ETT primarily manifesting as a genital tract fistula. The patient was initially misdiagnosed with vaginal carcinoma and had no history of gestational trophoblastic disease. This case aims to further explore the clinical characteristics and diagnostic challenges associated with this rare condition.

## Case presentation

**Figure 1:**
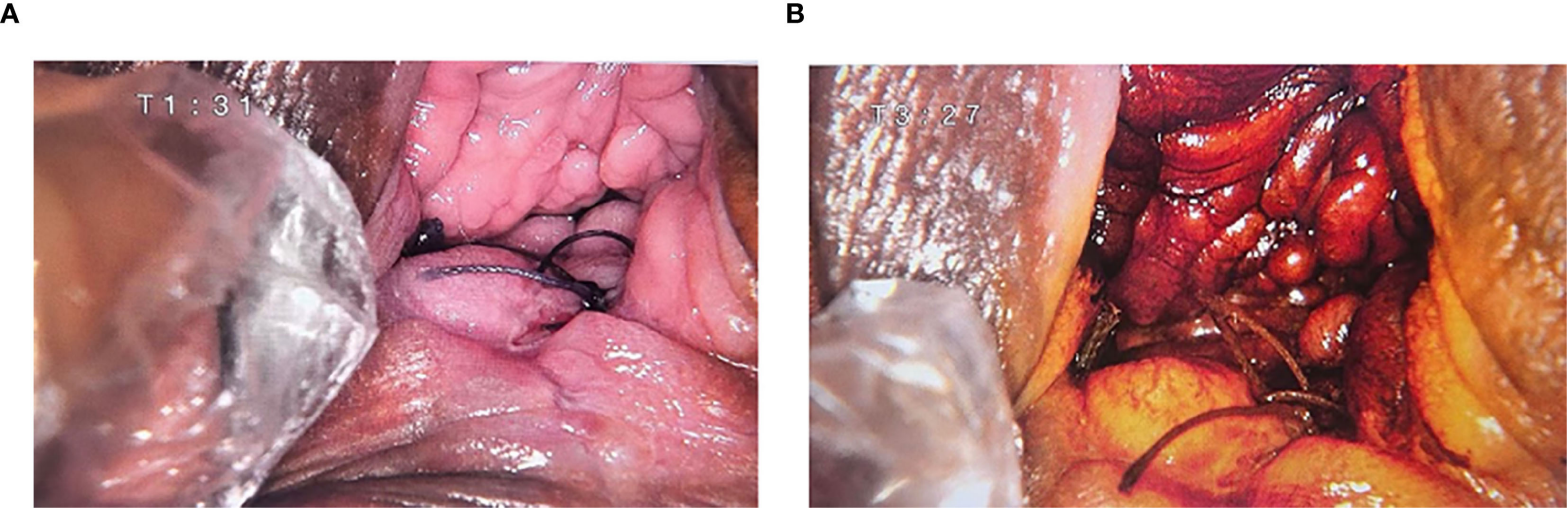
Colposcopy results. **(A)** Normal colposcopy image: A small opening and sutures are visible. **(B)** Colposcopy image after acetic acid test: A thin aceto-white area is visible in the posterior fornix.

**Figure 2:**
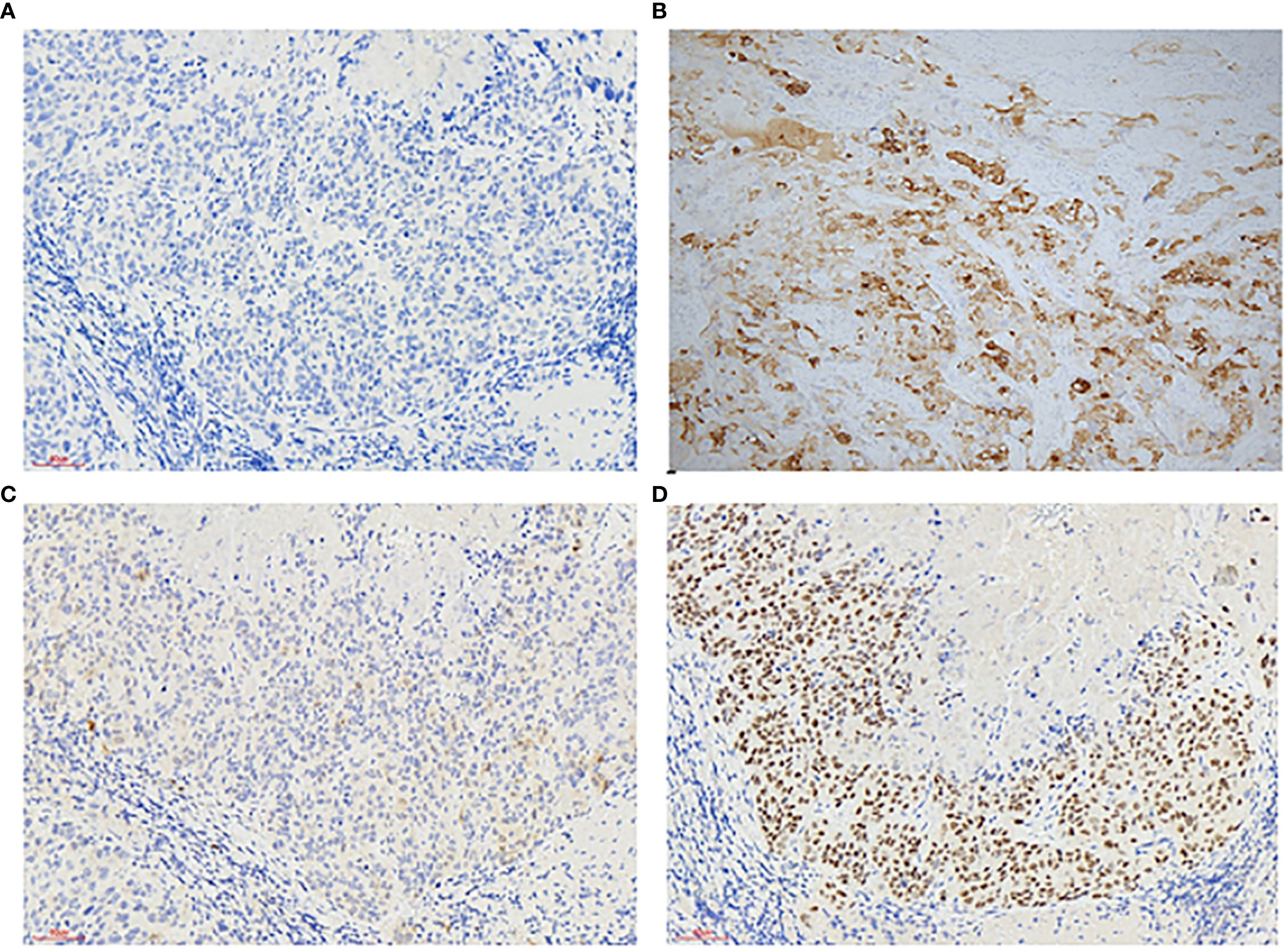
Immunohistochemistry of vaginal epithelial trophoblastic tumors: Immunohistochemical stains (200×) are positive for CK5/6 - **(A)** α-Inhibin + **(B)** P16 + **(C)** P63 + **(D)**.

A 50-year-old perimenopausal woman presented with a 3-year history of vaginal pain. She had previously undergone vaginal fistula repair at an external hospital, where a postoperative biopsy suggested vaginal carcinoma, leading to her referral to our institution for further management. Colposcopy revealed no significant pathological changes in the cervix, but sutures were noted near the vaginal orifice. Acetic acid testing showed no obvious abnormalities at the fistula site. The posterior perineal commissure appeared thickened with thin acetowhite epithelium, raising suspicion for possible lesions (**Figure 1**). To further clarify the diagnosis, a pathological consultation was conducted at our hospital. Immunohistochemical staining was negative for CK5/6 and positive for α-Inhibin, P16, and P63 (**Figure 2**), which are consistent with epithelioid trophoblastic differentiation. Tumor markers (CEA, CA125, SCC) are normal and serum hCG levels were within normal limits at <2 mIU/mL (reference range: <10 mIU/mL), in contrast to typical gestational trophoblastic neoplasms. Based on the patient’s pathological findings, the diagnosis was consistent with ETT.

The patient’s obstetric history included two vaginal deliveries of healthy infants in 1999 and 2000, with no history of hydatidiform mole or other gestational trophoblastic diseases. The patient opted to undergo total hysterectomy, bilateral salpingo-oophorectomy, partial vaginectomy, and resection of the posterior perineal commissure. Pathological examination of the uterus and adnexa revealed no lesions, confirming the diagnosis of a primary vaginal ETT, a rare entity with an unclear pathogenesis. Pathological findings from the partial vaginal and posterior perineal commissure tissue further supported the diagnosis of ETT. Notably, serum HCG levels remained normal throughout the course of the disease, and PET-CT showed no evidence of distant metastasis. Given the potential aggressiveness of ETT, the patient received 6 cycles of EMA-CO chemotherapy to reduce the risk of recurrence. The treatment plan is as follows:

Day 1 (EMA regimen):

Actinomycin D 500 μg intravenous infusion over 1 hour;
Etoposide 100 mg/m² intravenous infusion over 1 hour;
Methotrexate 100 mg/m² intravenous bolus;
Methotrexate 200 mg/m² intravenous infusion over 12 hours.

Day 2:

Actinomycin D 500 μg intravenous infusion over 1 hour;
Etoposide 100 mg/m² intravenous infusion over 1 hour;
Calcium folinate (leucovorin) 15 mg intramuscular injection, administered every 12 hours for 4 doses, starting 24 hours after the methotrexate intravenous bolus.

Day 8 (CO regimen):

Vincristine 2 mg intravenous bolus, administered 3 hours prior to cyclophosphamide;
Cyclophosphamide 600 mg/m² intravenous infusion over 2 hours.

Day 15: Initiate the next cycle starting with Day 1.

The patient tolerated chemotherapy well, with no severe adverse effects. At the 1-year follow-up, there was no evidence of distant recurrence or metastasis, and the patient remained in stable condition. The patient is also currently being followed up.

## Discussion

We herein present a clinically and academically critical case of primary vaginal ETT, which, to our knowledge, represents the first reported instance masquerading as a vaginal fistula. This unusual presentation underscores a notable diagnostic challenge, as a highly specialized malignant entity mimicked a benign gynecological condition—emphasizing the imperative for inclusion of ETT in the differential diagnosis of atypical vaginal lesions. ETT typically occurs in women of reproductive age, with an average onset at 36 years and peak incidence between 30 and 50 years. However, it can occasionally present in postmenopausal or perimenopausal women (7). The most common symptoms include abnormal vaginal bleeding and amenorrhea, though asymptomatic cases are rare. In this case, the patient was 50 years old and had experienced vaginal pain for 3 years. Her gynecological examination revealed only a small fistula at the inferior portion of the vagina is adjacent to the posterior perineal commissure. Given her clinical presentation, diagnosing ETT was particularly challenging. The differential diagnosis of ETT primarily includes cervical squamous cell carcinoma, placental site trophoblastic tumor (PSTT), choriocarcinoma, and epithelioid leiomyosarcoma. These conditions are typically distinguished based on histopathological features and immunohistochemical findings. The immunophenotype of ETT is characterized by both epithelial and trophoblastic markers (8, 9). Epithelial markers, such as P6CKpan, CK7, CK18, and EGFR, typically show strong and diffuse positivity, while EMA demonstrates moderate expression. In contrast, markers like CK5/6, CK34βE12, Cam5.2, and CEA exhibit weak or focal expression, with P16 typically being negative. Squamous cell carcinoma often shows strong expression of P16 and CK5/6. Trophoblastic markers, including HLA-G, P4-OHase, and CD10, generally show strong expression, with moderate positivity for α-Inhibin and weak or focal expression of HCG, hPL, and CD146. Notably, P63 is a key marker for distinguishing PSTT from ETT (10, 11). In this case, the patient exhibited positive expression of P63 and α-Inhibin, focal expression of P16, and negative expression of CK5/6, supporting the diagnosis of ETT.

To the best of our knowledge, this is the first reported case of primary vaginal ETT simulating a vaginal fistula. This conclusion is supported by a comprehensive literature review conducted using Boolean operators with the key terms “Epithelioid trophoblastic tumor” or “ETT” combined with “vagina” or “vaginal” across major databases including PubMed, Web of Science, and Embase, which identified only two previously reported cases of primary vaginal ETT, none of which were described with fistula-like features (**Table 1**). As indicated in **Table 1**, the typical manifestations of vaginal ETT include vaginal obstruction and pain. However, in this case, the patient primarily presented with vaginal pain accompanied by a vaginal fistula, without any signs of abnormal vaginal bleeding or obstruction. Based on current literature and the findings in this case, a direct diagnosis of ETT remains challenging due to its nonspecific symptoms and normal HCG levels. Nonetheless, vaginal delivery or induced abortion may be significant predisposing factors for the development of vaginal ETT. Ohira (12) suggested that episiotomy sites could also serve as a potential origin for primary vaginal ETT. Furthermore, Taliento, C reported that atypical epithelioid trophoblastic lesions, often accompanied by cysts and fistulas, may develop following cesarean section (14). Given the patient’s presentation of a genital tract fistula, a high index of suspicion for ETT is warranted in such cases.

**Table 1:** Summary of the clinicopathological characteristics of two cases of vaginal epithelioid trophoblastic tumor.

Study	Age	Symptoms	Childbearing history	Mode of delivery	HCG, mIU/mL	Immunostaining	Treatment	Follow up
Ohira, S, 2000 (12)	30	Vaginal tumor and pain	G1P1	Vaginal delivery (in 1999)	2.8	Positive for cytokeratin (CK22, CAM 5.2, CK18), inhibin-α, and Mel-CAM; focally positive for hPL, and negative for hCG, CD68, S-100, desmin, and vimentin; The Ki-67 index was approximately 15%.	Surgery	14 months
Jing Zhao, 2013 (13)	43	Vaginal tumor	G2P0 + 2	Induced abortions (in June 2007 and another in March 2009)	15.5	Positive for AE1/AE3, hCG, hPL, PLAP, P63, and CAM5.2; Negative for CEA, actin, melan-A, inhibin-α, HMB45, E-cadherin, and cyclin D1. The Ki-67 labeling index was approximately 15%	Chemotherapy and surgery. (Three courses of chemotherapy with a 3-week regimen of vincristine (VCR; 2 mg intravenously, day 1), floxuridine (FUDR; 800 mg/m2/day, intravenously, days 1–5), dactinomycin (Act-D, 200 kg/m2/day, intravenously, days 1–5), and etoposide (VP-16, 100 mg/m2/day, intravenously, days 1–5)	8 months

G, Gravida; P, Para; hPL, Human placental lactogen; PLAP, Placental alkaline phosphatase; P63, Tumor protein 63; CAM, Antibodies to cytokeratin; CEA, Carcinoembryonic antigen.

While choriocarcinoma is highly responsive to chemotherapy, ETT often exhibits significant chemoresistance, with surgical resection remaining the cornerstone of treatment (15). The management of primary vaginal ETT presents a significant challenge due to its extreme rarity and the consequent lack of evidence-based guidelines. The treatment strategy for our patient, comprising surgery followed by adjuvant EMA-CO chemotherapy, was formulated based on the established principles for high-risk gestational trophoblastic neoplasia (GTN) and ETT. It is important to acknowledge that other multi-agent regimens, such as FAEV (floxuridine, dactinomycin, etoposide, and vincristine) or EP-EMA (etoposide-cisplatin/etoposide-methotrexate-dactinomycin), are also employed in the treatment of high-risk GTN and could be considered valid alternatives for ETT. However, the superior efficacy and manageable toxicity profile of EMA-CO, coupled with our institution’s extensive experience with this protocol, guided our selection (16). Pelvic lymphadenectomy is typically not required for ETT; however, concurrent removal of uterine lesions is recommended if present. The efficacy of radiotherapy and chemotherapy in treating ETT remains uncertain. Studies suggest that the success rate of conservative treatment is approximately 20% (17). For cases of ETT not originating in the uterus, whether hysterectomy should be performed remains controversial. While the patient has achieved a 1-year disease-free interval—an encouraging early outcome—it is crucial to acknowledge that ETT is associated with a risk of recurrence beyond this period. Therefore, stringent long-term follow-up, including serial serum HCG monitoring and imaging, is mandated for this patient.

This report presents several strengths. Primarily, it constitutes, to the best of our knowledge, the first detailed documentation of a primary vaginal ETT masquerading clinically as a benign vaginal fistula. This unique presentation serves as a critical alert for clinicians, expanding the known clinical spectrum of ETT and potentially preventing diagnostic delay. Furthermore, the comprehensive description of the radiological and histopathological findings, including a complete immunohistochemical profile, provides a valuable reference for the diagnostic workup of similar rare cases. The successful application of a multimodal treatment strategy—combining surgery with adjuvant EMA-CO chemotherapy—also offers practical insights into the management of this elusive condition. However, our study is not without limitations. The inherent constraints of a single-case report limit the generalizability of our findings. Although the diagnosis of ETT is well-supported by histomorphology and immunohistochemistry (positive for α-Inhibin, p16, and p63; negative for CK5/6), additional staining for HLA-G and hPL was not performed to avoid imposing extra financial burden on the patient. While not essential for diagnosis in this case, inclusion of these markers could have provided further diagnostic confirmation. Finally, the relatively short follow-up period remains a constraint; given the documented potential for late recurrence in ETT, ongoing long-term surveillance of the patient is imperative.

## Conclusion

Vaginal ETT often presents with nonspecific clinical manifestations, which can lead to misdiagnosis. Symptoms such as vaginal discomfort, mild bleeding, or masses may closely resemble those of other gynecological conditions, complicating early identification. Vaginal delivery and induced abortion are recognized as potential risk factors. Clinically, in patients presenting with vaginal pain, masses, or genital tract fistulas, especially those with these risk factors, the possibility of a trophoblastic tumor should be strongly suspected. Timely evaluation and accurate diagnosis are crucial, as early detection plays a key role in improving prognosis.

## Data Availability

The original contributions presented in the study are included in the article material. Further inquiries can be directed to the corresponding author.
